# Caregiver Assistance with Young Children’s Emotion Regulation Strategies: Correspondence Between Global and Momentary Reports

**DOI:** 10.1007/s42761-025-00308-x

**Published:** 2025-06-06

**Authors:** Joanna H. Wright, Margaret N. Cox, Nicole R. Giuliani

**Affiliations:** 1 University of Oregon (Counseling Psychology and Human Services), Eugene, OR USA; 2 University of Oregon (Special Education and Clinical Sciences), Eugene, OR USA; 3 Gonzaga University (School of Education), Spokane, WA USA

**Keywords:** Emotion socialization, Emotion regulation, Ecological momentary assessment, Measurement, Early childhood

## Abstract

Caregivers play a central role in children’s emotion regulation development. Caregiver assistance with children’s deployment of specific emotion regulation strategies has gained attention in the literature as a key component of emotion socialization. Many studies have examined caregiver support for child emotion regulation strategies using global caregiver self-report measures, but few have leveraged ecological momentary assessment methods to learn about these momentary co-regulatory behaviors in daily life. Furthermore, the degree to which global and momentary reports correspond with each other remains unknown. Discrepancies between global versus momentary reports have important implications for research design and interpretation. The present study evaluated the degree to which caregiver reports of assistance with child emotion regulation strategies collected using ecological momentary assessment aligned with global, retrospective reports. Analyses focused on four emotion regulation strategies: acceptance, distraction, cognitive reappraisal, and expressive suppression. A USA sample of 174 primary caregivers with children ages 1–5.5 years completed online check-ins up to three times per day for seven days. Caregivers reported their child’s emotion and the emotion regulation strategies they helped their child use. Before they began the week of check-ins, caregivers completed a global measure of assistance with child emotion regulation strategies. Correlation and regression analyses showed evidence of correspondence between global and momentary reports for acceptance and expressive suppression, but not for distraction or cognitive reappraisal. The results caution against assuming global measures of caregiver assistance with child emotion regulations strategies uniformly reflect implementation in the context of daily parent–child interaction.

**Supplementary Information:**

The online version contains supplementary material available at 10.1007/s42761-025-00308-x.

Parents and other primary caregivers (“caregivers”) provide foundational support for children’s emotion regulation (ER) development. Caregiver ER assistance is crucial in early childhood, when children’s ER systems develop rapidly (Chronis-Tuscano et al., 2022; Eisenberg et al., 2010; Harrington et al., 2020; Hostinar et al., 2014; Kerr et al., 2019). Early ER skills enable children to manage emotions and behavior and engage adaptively with their social environments (Morris et al., 2007), which in turn supports socioemotional competence and school readiness (Harrington et al., 2020).

Caregiver assistance with children’s use of specific ER strategies has gained attention in the literature as a key element of emotion socialization (Cohodes et al., 2022). According to the Process Model of Emotion Regulation, ER occurs across temporal stages (i.e., situation selection, situation modification, attentional deployment, cognitive change, response modulation), each of which contains multiple ER strategies an individual may employ to alter the course of an unfolding emotion (Gross, 1998, 2015). Young children learn to navigate ER stages and strategies with help from their caregivers. For example, a caregiver may help their child practice acceptance, a cognitive change strategy, by nonjudgmentally acknowledging and affirming the child’s feelings. Or they may support their child’s use of distraction, an attentional deployment strategy, by redirecting the child’s attention away from a stressor toward a comfort item. Alongside other forms of emotion socialization, these co-regulatory behaviors contribute to children’s understanding, experience, expression, and regulation of emotion (Cohodes et al., 2022; Eisenberg, 1998; Morris et al., 2007).

Most studies of caregiver support for child ER have relied on global surveys of caregiver attitudes, beliefs, and behaviors regarding child emotion. However, retrospective reports “collapsed across time and place” (Silk, 2019, p. 2009) may lack contextual information such as the specific emotion being regulated or caregivers’ real-time regulation goals. Alternatively, ecological momentary assessment (EMA) methods can improve ecological validity and capture short-term temporal dynamics (Heron et al., 2019; Shiffman et al., 2008; Silk, 2019). These strengths are valuable for ER and emotion socialization research because of the short time scales in which many affective caregiver-child interactions unfold (Buhler-Wassman & Hibel, 2021; Heron et al., 2019).

Despite the benefits of EMA methods, little is known about how caregivers support their child’s use of ER strategies in daily life and how this corresponds with what caregivers report globally. Correspondence studies of related constructs have yielded mixed results. Koval et al. (2022) and McMahon and Naragon-Gainey (2020) studied correspondence of global and momentary reports for adults’ intrinsic (“self-focused,” Petrova & Gross, 2023) ER, finding that correspondence was relatively weak overall and varied between ER strategies. McMahon and Naragon-Gainey, (2020) concluded that, although global ER measures may broadly index tendency to use multiple ER strategies, they may not capture implementation of specific strategies in daily life. Whether these findings generalize to caregiver-child extrinsic (“other-focused,” Petrova & Gross, 2023), ER remains unknown.

The present study aimed to address these gaps by (1) using momentary (EMA) reports to gain a rich picture of caregivers’ assistance with children’s use of ER strategies in daily life and (2) examining correspondence between momentary and global reports of caregiver assistance with children’s ER strategies. Aim 2 focused on four ER strategies in response to negatively-valenced emotions: acceptance, distraction, cognitive reappraisal, and expressive suppression. These have been highlighted by prior literature as key ER strategies (McRae & Gross, 2020) and were selected based on conceptual alignment between our momentary and global measures.

Because existing literature regarding caregiver scaffolding of child ER is limited, we took an exploratory approach to these research questions. With Aim 1, we sought to describe caregivers’ assistance with children’s use of ER strategies in daily life. With Aim 2, we sought to explore alignment between global and momentary reports of caregiver assistance with child ER strategies using regression analyses.

## Method

### Participants

The original sample consisted of 198 primary caregivers across the USA. *A priori* power analyses calculated using G*Power 3.1 (Faul et al., 2009) indicated that this would provide adequate power to address the present aims using multiple regression models. A sample size of at least 114 was required to be sufficiently powered at 1-*β* = .80 to identify medium effect sizes (*f*^2^ = 0.15) with five covariates and four predictors (Aim 2) at an alpha of .05. Model specifics are described below (see Data Analyses).

Primary caregivers of children ages 18 months to 5 years were invited to participate. Eligibility required participants to have had at least 50% custody of the focal child for the last year and read English fluently. If caregivers had multiple children in the target age range, they were instructed to answer questions with regard to one of their children.

### Recruitment

The study team used online flyers and video to recruit participants remotely from across the United States. A convenience sampling and snowball recruitment approach used social media and direct outreach to community organizations and parenting groups. Recruitment began in May 2022 and data collection ended in October 2022.

### Procedure

All study activities including recruitment, eligibility screening, intake measures, and EMA check-ins, took place remotely. An initial survey screened for participant eligibility and described study protocols and compensation. Participants gave informed consent via electronic signature acknowledging understanding of study purpose, duration, risks, benefits, alternatives to participation, and compensation. Consenting participants received a video and flier with instructions about EMA procedures and were encouraged to reach out to the researchers via email or social media if they had questions.

Full participation included completing an approximately 20-min intake survey with demographic information, the Parental Assistance with Child Emotion Regulation scale (PACER) (Cohodes et al., 2022), and the Integrative Child Temperament Scale (ICTS) (Zentner, 2020; Zentner & Wang, 2013), and other questionnaires not included in these analyses. Upon completion of intake measures, eligible participants were invited to begin EMA data collection.

The EMA portion of the study utilized a time-based sampling design (Shiffman et al., 2008), sending prompts to participants’ cell phones three times per day (11 am, 3 pm, and 7 pm local time) for 7 consecutive days. Participants were encouraged to complete the check in as soon as possible after receiving the prompt and had to complete it within 1 h to earn compensation. These 2-min check-ins prompted participants to think about the prior 4 h and report their strongest emotion, their child’s strongest emotion, their responses to their child’s strongest emotion, and other questions about the caregiving context. Participants could earn up to $56 in reimbursement depending on the number of check-ins they completed. To screen out bot responses and ensure data quality, participants were contacted by phone/text with confirmation questions, and EMA check-ins included attention check questions. The protocol for this study was approved by the University of Oregon Institutional Review Board (IRB).

### Measures

#### Momentary Report of Assistance with Child Emotion Regulation Strategies

At each EMA check-in, participants were asked to report the strongest emotion their child felt in the prior 4 h from a list of 19 common emotion words (joyful, angry, accomplished, irritable, grateful, worried, content, stressed, strong, sad, proud, lonely, interested, hopeless, excited, guilty, attentive, frustrated, empty; Kerr et al., 2021) and the intensity of the emotion on a 1–6 scale from 1 (*very negative*) to 6 (*very positive*). Then they were asked, “How did you respond to your child’s emotion?” and presented with 10 ER strategies. Our research team developed this question and adapted response options from Grommisch et al. 2020 to be accessible to culturally diverse caregivers of young children (Online Resource 1, Table 1). Participants could select as many ER strategy options as they wanted at each check-in. Table 1 displays the four items used in the present study.

**Table 1:** Momentary caregiver assistance with Child ER strategies

ER strategy	Item
Acceptance	I expressed that it was OK to feel their emotion
Cognitive reappraisal	Offered them ways to interpret the situation (e.g., explained reasoning)
Distraction	I encouraged them to do something pleasant (e.g., watch cartoons)
Expressive suppression	I verbally encouraged them to change their emotion (e.g., “don’t cry”)

This momentary measure of caregiver assistance with child ER strategies was scored by summing the number of times each participant endorsed the given strategy and dividing by the total number of EMA check-ins they completed, providing a frequency score from 0–1 for each person. This approach to bringing observation-level data to the person-level is an established means of leveraging EMA data to gain overall information about daily, contextually-situated experience that is not time-point contingent (Heron et al., 2019).

#### Global Report of Assistance with Child Emotion Regulation Strategies

The Parental Assistance with Child Emotion Regulation (PACER) assesses caregiver support for child use of emotion regulation strategies drawn from the process model of emotion regulation (Cohodes et al., 2022). PACER items begin with the statement “When my child is having negative feelings…” and end with different strategies (i.e., “I help my child take steps to solving a problem.”). Respondents rate their likelihood of engaging in that strategy from 1 (*strongly disagree*) to 7 (*strongly agree*). Each item corresponds to one of 10 ER strategies: acceptance, reappraisal, avoidance, behavioral disengagement, distraction, expressive suppression, problem solving, rumination, social support search, and venting. The original validation study supported the 10-factor structure and showed strong internal consistency, acceptable test–retest reliability, and acceptable convergent validity. A follow-up validation study with caregivers of children ages 3 months to 5.5 years also found support for the 10-factor structure (Mancini et al., 2022). The present study used four PACER subscales: acceptance, distraction, cognitive reappraisal, and expressive suppression. Cronbach’s alphas in the present sample showed very good to excellent internal consistency for acceptance (*α* = 0.89), distraction (*α* = 0.96), cognitive reappraisal (*α* = 0.92), and expressive suppression (*α* = 0.92).

#### Child Temperament

The Integrative Child Temperament Screener (ICTS) was used to assess aspects of child temperament related to caregiver emotion socialization behaviors (Zentner & Wang, 2013; Zenter, 2020). We used the ICTS lack of impulse control composite comprised of the frustration proneness (e.g., “Cries or yells when asked to stop favorite occupation”) and reversed attentional control scales (e.g., “When looking at a book or painting, is quickly bored and changes activity” (Zentner & Wang, 2013; Zenter, 2020). ICTS items are scored from 1 (*the behavior occurs never or hardly ever*) to 6 (*the behavior occurs always or close to always*).The ICTS has shown adequate test–retest and interrater reliability, criterion validity with measures of behavior problems, and convergent validity (Zentner, 2020). Reliability for the lack of impulse control composite in the present study sample was good (*α* = .75).

#### Demographic Information

At intake, caregivers reported gender (*male*, *female*, *nonbinary*, *gender fluid*, *prefer not to say*), age, and ethnicity for themselves and their child. They also reported how many years of education they had completed, household annual income in dollars, and their relationship to their child (e.g., mother, father, stepparent).

### Data Cleaning

The original sample consisted of 2,930 completed EMA check-ins from 198 participants. Automated and manual cleaning removed check-ins that were duplicates due to technical error, completed outside of the expected data window, or suggested evidence of backfilling (completed within 1 h of that participant’s previous check-in). None of these steps resulted in exclusion of any participants but decreased the number of check-ins to 2,744.

After the above cleaning steps were conducted, the compliance rate for the sample was 72.97% (*SD* = 31.7%), with participants completing an average of 15.32 out of 21 total possible check-ins. The 21 participants with very poor compliance (< 20% of check-ins completed) were excluded from analyses. Of the remaining 177 participants, 63.7% had compliance rates of 75% or above. There were no significant differences between included and excluded participants with regard to caregiver race, caregiver gender, child gender, or income, but the proportion of caregivers with lower education levels was significantly larger among excluded participants (*χ*^2^ = 19, df = 4, *p* < .001).

Finally, the analytic sample was limited to only check-ins in which caregivers reported being with their child in the time since the previous check-in (2,561 check-ins) and check-ins in which the caregiver reported a child emotion from the previous 4 h (2,541 check-ins). To align with the PACER which measures responses to child negative emotion, only check-ins in which caregivers reported a child negative emotion in the previous 4 h (i.e., angry, irritable, worried) were included in analyses (829 check-ins). This decreased the analytic sample from 177 to 174 participants.

### Data Analyses

Analyses were conducted in R Studio Version 3.0 using the using the *dplyr* (Wickham, François et al., 2021; Wickham, Hester et al., 2021), *stringr* (Wickham, 2019), *tidyverse* (Wickham et al., 2019), *psych* (Revelle, 2023), *naniar* (Tierney et al., 2021), *tidyr* (Wickham, 2021), *sjPlot* (Lüdecke, 2023), *devtools* (Wickham, François et al., 2021; Wickham, Hester et al., 2021) and *broom* (Robinson et al., 2021) packages. We first described frequencies of caregiver momentary assistance with child ER strategies. We then ran bivariate correlations to assess correspondence between momentary and global reports. Spearman’s rank correlation was used because momentary reports were non-normally distributed (Schober et al., 2018). Scatter plots showed that all four variable pairs (i.e., momentary acceptance with global acceptance) met the assumption of a monotonic relationship between variables (Conover & Iman, 1981). *P*-values were adjusted for multiple comparisons using the Benjamini–Hochberg method (Benjamini & Hochberg, 1995).

Next, we used multiple linear regressions to test relationships between global and momentary reports while controlling for child age, child and caregiver gender (1 = female, 0 = not female), caregiver education level (years completed), and child temperament (ICTS lack of impulse control scale), based on prior research showing effects of these factors on emotion socialization behaviors (Baker et al., 2011; Cunningham et al., 2009; Kiff et al., 2011; Lunkenheimer et al., 2020; McKee et al., 2021; Morris et al., 2007). All models also included all momentary ER strategies as independent variables, because it is possible that individuals who tend to help their child with one ER strategy may also tend to help their child with multiple strategies. Regressing all momentary strategies onto each global measure helped evaluate whether global reports were uniquely associated with their corresponding momentary report, holding constant any general tendencies to help their child with multiple ER strategies in daily life. Person-level compliance percentages were added to each model using the *lm() weights* function so that participants with higher compliance percentages were given more weight. Following checks of regression assumptions, models were rerun with Cook’s Distance outliers removed, which improved diagnostic plots and did not substantially alter results. A conservative approach was taken by removing model-specific outliers for final analyses. No differences were found between outliers and non-outliers in demographic or study variables. VIF analyses did not indicate multicollinearity (VIF values ranged from 1.0 to 1.2).

Identification of true discrepancies between global and momentary measures requires items that are relatively conceptually and semantically aligned (Rosenbusch et al., 2020). Therefore, examination of similarity of item wording for global and momentary pairs was a key methodological consideration. We assessed semantic similarity quantitatively with item-level correlations and cosine (Online Resource 1, Tables 2–3) and qualitatively (subjective review of item wording). Although conceptual fit guided measure selection in this study, similarity of wording for global and momentary measures varied somewhat among scales. This information was set aside to be used in interpretation of results.

**Table 2:** Demographic characteristics

Characteristic	Caregiver					Child		
	*n*	%	*M*	*SD*	*n*	%	*M*	*SD*
Age			34.2	5.7			3.05	1.12
Gender								
Man/boy	7	4.1			92	54.4		
Woman/girl	161	95.3			74	43.8		
Other	1	0.5			3	1.8		
Race/ethnicity								
American Indian	1	0.5			3	1.8		
Asian	5	2.9			7	4.1		
Black	13	7.7			14	8.3		
Hispanic/Latino	2	1.2			4	2.4		
White	143	84.6			145	85.8		
Mixed	4	2.4			24	14.2		
Other	5	2.9			1	0.5		
Education level								
High school/GED	17	10.1						
Associate degree	27	16						
Bachelor’s degree	74	43.8						
Master’s degree	41	24.3						
Doctorate degree	7	4.1						
Household income ($)			113,652	83,704				

Caregivers could select multiple options for their race and their child’s race

**Table 3:** Child emotions across observations (*n* = 2,541)

Child emotion	*n*	*%*	Totals	
			*n*	*%*
Accomplished	57	2.2	1,712 positive emotions	67.4% positive emotions
Attentive	54	2.1		
Content	288	11.3		
Excited	353	13.9		
Grateful	8	0.3		
Interested	166	6.5		
Joyful	703	27.7		
Proud	55	2.2		
Strong	28	1.1		
Angry	106	4.2	829 negative emotions	32.6% negative emotions
Empty	3	0.1		
Frustrated	229	9.0		
Guilty	11	0.4		
Hopeless	5	0.2		
Irritable	200	7.9		
Lonely	23	0.9		
Sad	155	6.1		
Stressed	44	1.7		
Worried	53	2.1		

## Results

### Sample Demographics

Table 2 presents demographic characteristics for the analytic sample (*n* = 174). Caregivers in this sample resided in 35 states across the United States. Most caregivers identified as a woman (95.3%) and identified their child as a boy (54.4%) or girl (43.8%). Most caregivers identified as White (84.6%) and identified their child as White (85.8%) or mixed race (14.2%). Mean caregiver age was 34.2 years (*SD* = 5.65, range 23–65). Average child age was 3.05 years (*SD* = 1.12, range 1.5–5). Highest level of education completed varied, with 26.1% holding a high school/GED degree or associate degree, 43.8% holding a Bachelor’s degree, and 24.3% holding a Master’s degree. Mean household annual income was $113,652 (*SD* = $83,704, range $18,000–$500,000).

### Aim 1: Describing Caregivers’ Assistance with Child ER Strategies

Table 3 provides context for the results to follow by describing the frequency with which caregivers reported specific child negatively-valenced emotions. Caregivers reported child negative emotion in approximately one third of all check-ins. Among all check-ins, caregivers most often reported child positive emotion as joyful (27.7%), excited (13.9%), and content (11.3%), and child negative emotion as frustrated (9%), irritable (7.9%), sad (6.1%), and angry (4.2%). At the participant level, average frequency of child negative emotions was 4.8 check-ins (*SD* = 3.3, range 1–18).

Table 4 presents means and standard deviations for all study variables. In response to child negatively-valenced emotions, caregivers reported supporting their child with acceptance, cognitive reappraisal, distraction, and expressive suppression in 47%, 42%, 22%, and 12% of their momentary check-ins, respectively. Global PACER scores ranged from 5 to 35 and were the for highest for acceptance (*M* = 30, *SD* = 5), followed by cognitive reappraisal (*M* = 26, *SD* = 5.9), distraction (*M* = 24, *SD* = 7.2), and expressive suppression (*M* = 11, *SD* = 6.2).

**Table 4:** Means, standard deviations, and zero-order correlations of study variables

Variable	*M (SD)*	1	2	3	4	5	6	7	8	9	10	11	12
1. EMA acceptance	.47 (.37)												
2. EMA distraction	.22 (.26)	.02											
3. EMA reappraisal	.42 (.34)	.23*	.08										
4. EMA suppression	.12 (.21)	−.28**	.15	.12									
5. PACER acceptance	30 (5)	.31***	.00	.04	−.28***								
6. PACER distraction	24 (7.2)	−.03	**.12**	.11	.13	−.02							
7. PACER reappraisal	26 (5.9)	−.01	−.05	**.04**	−.01	.22	.45***						
8. PACER suppression	11 (6.2)	−.20	−.01	.02	.30**	−.40***	.30**	.11					
9. Child age	3.1 (1.1)	−.17	−.26***	−.07	.01	−.04	−.05	.14	.05				
10. Child gender^a^	.43	.00	−.08	−.10	−.09	.00	−.04	−.08	−.04	−.02			
11. Caregiver gender^a^	.96	−.12	.06	.06	−.08	−.09	−.08	−.04	−.04	−.06	.08		
12. Child LOIC	19 (5.1)	−.02	.12	.02	.01	−.04	.05	−.10	−.05	−.13	−.12	−.03	
13. Caregiver education	15.92	.04	−.17	.12	−.02	−.02	.00	.01	−.03	−.04	.04	.00	−.06

*EMA* ecological momentary assessment, *PACER* Parental Assistance with Child Emotion Regulation (Cohodes et al., 2022). Reappraisal, cognitive reappraisal; suppression, expressive suppression. Child LOIC, Child Lack of Impulse Control (Integrative Child Temperament Scale, Zentner, 2020). Caregiver education, years completed. Correlations among EMA and PACER scales (1–8) are corrected for multiple comparisons using Benjamini–Hochberg method (Benjamini & Hochberg, 1995). Correspondence pairs are indicated in bold

^a^1 = female, 0 = not female

*p <.05, **p <.01, ***p <.001

For context, Table 5 reports momentary results for all ER strategies, including those not analyzed in this study. In addition to acceptance and cognitive reappraisal, caregivers also frequently reported helping their child with labelling of negatively-valenced emotions.

**Table 5:** Frequencies of momentary caregiver assistance with ER strategies

	Observation-level		Person-level average
ER strategy	*n*	*%*	*%*
Acceptance	359	16%	47%
Cognitive reappraisal	349	16%	42%
Distraction	205	9%	22%
Expressive suppression	131	6%	12%
Distraction (avoidant)	169	8%	20%
Ignoring	32	2%	4%
Labeling	345	16%	41%
Situation modification	216	9%	26%
Social sharing	233	11%	28%
No regulation	166	8%	20%

Data represent responses to child negative emotion only. Caregivers were able to check multiple ER strategies at each check-in

To further describe caregiver support for child use of ER strategies in daily life, we examined whether momentary reports for each ER strategy varied by child age and child gender. T-tests showed that caregivers’ use of each specific strategy did not vary significantly by child gender. Linear regression showed that child age was negatively associated with caregiver support for child acceptance, *β* = − 0.07, *SE*(*β*) = 0.02, *t*(167) = − 2.78, *p* < .001. Child age was also negatively associated with caregiver support for child distraction, *β* = − 0.05, *SE*(*β*) = 0.02, *t*(167) = − 3.42, *p* < .001. Caregiver support for child cognitive reappraisal and expressive suppression did not vary significantly by child age.

### Aim 2: Correspondence of Momentary and Global Reports of Caregiver Assistance with Child ER Strategies

Bivariate correlations assessed correspondence between momentary and global reports (Table 4). Momentary acceptance was moderately, positively associated with global acceptance, *r* = .31, *p* < .001 and momentary expressive suppression was moderately, positively associated with global expressive suppression, *r* = .30, *p* < .001. Momentary and global distraction and cognitive reappraisal were not significantly associated with their corresponding global measure.

To further examine these relationships, four multiple linear regression models were tested, one for each of the four global reports of caregiver emotion regulation strategy use as the dependent variable (Online Resource 1, Tables 4 − 7). We also used semi-partial correlations to estimate effect size, which can be interpreted like a correlation coefficient and aid interpretability of results. Momentary acceptance was significantly associated with global acceptance, *sr* = 0.26, *p* < 0.01. Momentary expressive suppression was significantly associated with global expressive suppression, *sr* = 0.28, *p* < 0.01. Momentary cognitive reappraisal and momentary distraction were associated with their global counterparts in the expected direction, but these effects were not significant. Correcting for multiple comparisons using the Benjamini–Hochberg procedure (Benjamini & Hochberg, 1995) did not alter results. Re-running regression models with the full sample, including the 21 participants with low EMA compliance, also did not change the significance of results.

## Discussion

The first aim of this study was to describe caregiver support for child ER strategy use in daily life. We found that caregivers reported child negatively-valenced emotions infrequently (20% of check-ins). Caregivers often responded to negatively-valenced emotions by helping the child acknowledge their emotion (acceptance) or altering the meaning of a situation to modify the emotional experience it elicits (cognitive reappraisal). Consistent with prior research, caregivers in this sample rarely reported encouraging their child to suppress negative emotion (Cohodes et al., 2022; Mancini et al., 2022). Further descriptive analyses showed that caregiver support for child acceptance of negative emotion and distraction from negative emotion decreased with child age. This finding builds on prior research showing variation in caregiver responses to child emotion across developmental stages. McKee et al. (2021) studied emotion socialization in families with children ages 3 − 12 and found that parents of younger children were more likely to be in the “emotion coaching” profile and parents of older children were more likely to be in the “emotion dismissing” profile. From a developmental perspective, factors underlying children’s ER skills (i.e., executive function, inhibitory control) develop rapidly in the early years (Gross & Cassidy, 2019), so caregivers may adjust ER strategy use as their child grows. Variation in caregiver assistance with child ER strategy use by age warrants further investigation.

The second aim of this study was to test the degree to which momentary reports of caregiver assistance with child ER correspond with global reports. Regression analyses showed small, significant semi-partial correlations between momentary and global reports for acceptance (*sr* = .26) and expressive suppression (*sr* = .28), but no such relationship for distraction or cognitive reappraisal.

### Global-Momentary Correspondence for Expressive Suppression

The small, positive association between global and momentary reports of expressive suppression observed in this study aligns with prior research. McMahon and Naragon-Gainey, 2020 found stronger correspondence between momentary and global reports for expressive suppression than for most other strategies assessed. Prior work suggests that the tendency to use expressive suppression to regulate emotion may be less contextually variable than the tendency to use other, less suppressive strategies (Aldao & Nolen-Hoeksema, 2012). Among our sample, it may be that caregiver’s global reports of expressive suppression were consistent with momentary reports because expressive suppression in daily life was relatively unaffected by situational factors like the specific emotion being regulated or the intensity of the emotion.

### Acceptance and Expressive Suppression: Opposite Ends of a Continuum

As with expressive suppression, we also found a small, positive association between global and momentary reports of acceptance. The similar levels of global-momentary correspondence for acceptance and expressive suppression may reflect the nature of these constructs as opposite ends of a single continuum (see Naragon-Gainey et al., 2017). Prior research has shown that caregivers fall into either an “emotion coaching” profile (acknowledging, labeling, and accepting children’s emotions) or an “emotion dismissing” profile (distracting, ignoring, dismissing, or suppressing children’s emotions) (Frogley et al., 2023; Gottman et al., 1996). In the present study, caregivers who frequently helped their child accept negative emotion rarely encouraged their child to suppress negative emotion. This pattern was evident within and across measurement modalities (Table 4; correlation between momentary acceptance and momentary expressive suppression: *r* = − .28, *p* < .01; correlation between global acceptance and momentary expressive suppression: *r* = − .28, *p* < .001).

### Cognitive Reappraisal and Distraction: Unique Measurement Considerations

The absence of a significant relationship between momentary and global measures of cognitive reappraisal aligns with prior research. Koval and colleagues’ study of adult intrinsic, self-focused ER (2022) found weaker global-momentary correspondence for cognitive reappraisal than for most other ER strategies. They suggested this may be because cognitive reappraisal is a more nuanced, covert process than overt behavioral strategies like expressive suppression. Reappraisal requires substantial cognitive resources (McRae et al., 2012; Petrova & Gross, 2023), which may limit caregivers’ accurate identification of their use of this strategy in global and/or momentary reports.

Alternatively, discrepancies between global and momentary reports of cognitive reappraisal could indicate flexible, adaptive implementation of this strategy in daily life (Aldao & Nolen-Hoeksema, 2012). Regulatory flexibility involves adjusting one’s regulatory goals based on contextual factors such as emotional intensity and situational affordances (Petrova & Gross, 2023). In the present study, caregivers who endorsed high levels of cognitive reappraisal globally may have had greater knowledge of this strategy and thus determined that it was not a developmentally appropriate response in the contexts sampled during the EMA period. This knowledge, combined with regulatory flexibility, may have enabled them to identify and implement other strategies that they perceived to best fit their child’s situational ER needs.

Furthermore, caregiver momentary support for child cognitive reappraisal may be difficult to assess via EMA methods. Cognitive reappraisal is understood to be “multifaceted” (Koval et al., 2022, p. 13), with subcomponents that are difficult capture using single-item EMA measures. The difficulty of measuring adult intrinsic, self-focused cognitive reappraisal likely extends to caregiver support for child cognitive reappraisal as well. Weaker construct validity of a single-item EMA measure of caregiver support for child cognitive reappraisal may have contributed to discrepancies.

As with cognitive reappraisal, results showed no evidence of correspondence for global and momentary distraction. Sources of this discrepancy warrant further investigation. Notably, PACER validation research by Cohodes et al. 2022 showed lower test–retest reliability over 1 week for distraction than for most other ER strategies. Our results affirm the need for further research into variability in caregivers’ implementation and reporting of this ER strategy.

### Semantic Considerations

Between-scale variation in semantic similarity of EMA items and their global counterparts may have influenced results. Previous ER correspondence research has noted the importance of conceptual alignment (Herbers et al., 2017; McMahon & Naragon-Gainey et al., 2020), and conceptual fit guided measure selection in the present study. Quantitative and qualitative assessment of semantic similarity showed that some global items were worded more similarly to their EMA counterpart than others. For example, the PACER acceptance item “I help my child understand that it’s okay to have negative feelings” shares some words with the EMA item “I expressed that it was okay to feel their emotion,” and showed a significant, positive relationship in item-level correlational analyses. However, the degree to which semantic similarity mapped onto strength of correspondence varied (Online Resource 1, Tables 2–3). Considering alignment of findings with prior research, it is unlikely that results are due to semantic similarity alone.

#### Implications for Theory: Applying the Accessibility Model to Emotion Socialization

Robinson and Clore’s (2002) accessibility model of emotion self-report posits that global versus momentary reports draw from different sources of information. EMA reports draw from highly contextual but fleeting information that is not readily encoded in memory, while global reports draw on more abstract conceptualization of the self (Robinson & Clore, 2002). The results of the present study affirm prior research suggesting that this theory applies to narrow constructs such as emotion regulation (Koval et al., 2022) and, as we add here, caregiver support for some child ER strategies.

#### Limitations and Future Directions

Limitations of the present study should be considered when interpreting results. First, recruitment was limited to English-speaking caregivers, and despite efforts to recruit a diverse sample, most caregivers identified as White and female. This limits generalizability of results, particularly given the culturally-situated nature of emotion socialization (Eisenberg, 2020). Most caregivers reported low levels of child negative affect, so results may not generalize to caregivers of children who experience more frequent or more intense negative affect. Second, momentary caregiver support for child ER strategy use was measured using single items created for this study. This is common in EMA studies of ER (Koval et al., 2022) but future research should use validated EMA scales if available (i.e., Medland et al., 2020). Relatedly, the construct validity of the EMA item should be considered. The question stem (“How did you respond to your child’s emotion?”) was developed by our research team to ask caregivers how they respond to their child’s emotion by helping their child with ER strategies. Drawing on emotion socialization (Eisenberg et al., 1998) and emotion regulation (Gross, 1998, 2015) frameworks, we approached caregiver responses to child emotion and caregiver assistance with child ER strategies as distinct yet related constructs (Cohodes et al., 2022). Caregivers respond to child emotion in numerous ways, including, but not limited to, supporting their child with use of an ER strategy. Furthering tailoring and validating an EMA item specifically about caregiver scaffolding of child ER strategies could strengthen future work in this area. Third, EMA and global measures were both self-reports. Social desirability can compromise the validity of parent self-reports of parenting behavior (Morsbach & Prinz, 2006); in particular, this may have contributed to low levels of expressive suppression in global and momentary reports. Fourth, between-scale variation in semantic similarity of EMA items and their global counterparts may have influenced results. Future research should design EMA items to align as closely as possible to their global counterpart. Fifth, future research can extend this work by exploring caregiver support for child use of multiple ER strategies in a single regulatory episode (“polyregulation”, Ford et al., 2019), and whether this affects correspondence between global and momentary ER measures. Our momentary measure of ER strategy use did not consider how many ER strategies caregivers used at a given timepoint. Caregivers may often assist their young child with several different ER strategies simultaneously or in close succession, particularly in situations when caregivers attempt several strategies before one is successful for meeting their regulation goals. Finally, the present analyses did not examine other factors that influence self- and other-focused ER, including emotion beliefs (Petrova et al., 2023), emotion recognition (Gottman et al., 1996), and caregivers’ own emotions and ER skills (Hajal & Paley, 2020).

Despite these limitations, the present study offers novel insight into emotion socialization practices by describing caregiver assistance with child ER strategies in daily life. It also evaluates correspondence between momentary and global reports of caregiver assistance with child ER strategies, addressing a key gap in the literature. Strengths of this research include a large and geographically diverse sample, transparent reporting of missing data, and examination of semantic similarity as a potential confound in analyses. Results showed relatively small, yet significant associations of momentary acceptance and expressive suppression with their global counterparts, but no such evidence of correspondence for cognitive reappraisal and distraction. These results caution against assuming global measures of caregiver support for child ER uniformly reflect implementation in daily life. Further research should assess sources of discrepancies to inform measure selection and interpretation in emotion socialization research.

## Supplementary Information

Below is the link to the electronic supplementary material.

ESM 1 (DOCX 35.0 KB)

## Additional Information

### Data availability

Data and code are available at https://osf.io/xu7rt/.
