# Supplementary material for: Caregiver Assistance with Young Children’s Emotion Regulation Strategies: Correspondence Between Global and Momentary Reports
Source: Affect Sci. 2025 Jun 6;6(3):403–13. doi: 10.1007/s42761-025-00308-x (PMC12579597; doi:10.1007/s42761-025-00308-x)
Supplement: Supplementary file 1 — (DOCX 35.0 KB) [file 42761_2025_308_MOESM1_ESM.docx]

**ONLINE RESOURCE 1**

Article Title: Caregiver Assistance with Young Children’s Emotion Regulation: Correspondence between Global and Momentary Reports

Journal: Affective Science

Authors: Joanna H. Wright^a^, Margaret N. Cox^b^, and Nicole R. Giuliani^b^

Author Affiliations:

Joanna H. Wright^a^, Margaret N. Cox^bc^, and Nicole R. Giuliani^b^

^a^ University of Oregon (Counseling Psychology and Human Services), Eugene, OR, USA

^b^ University of Oregon (Special Education and Clinical Sciences), Eugene, OR, USA

^c^ School of Education, Gonzaga University, WA, USA

Correspondence to Joanna Wright at [jwrigh23@uoregon.edu](mailto:jwrigh23@uoregon.edu).

**Table 1**

*Momentary Caregiver Assistance with Child ER Strategy (All Items)*

| ER Strategy | Item |
| --- | --- |
| Acceptance | I expressed that it was OK to feel their emotion |
| Cognitive Reappraisal | Offered them ways to interpret the situation (e.g., explained reasoning) |
| Distraction (Positive) | I encouraged them to do something pleasant (e.g., watch cartoons) |
| Distraction (Avoidant) | I encourage them to do something more productive (e.g., pick up toys) |
| Expressive Suppression | I verbally encouraged them to change their emotion (e.g., “don’t cry”) |
| Ignoring | I ignored their behaviors or feelings |
| Labeling | I verbally provided a label for the emotion (e.g., “you’re feeling sad” |
| Situation Modification | I physically changed the situation (e.g., hid broken toy so they couldn’t see it, removed child from environment) |
| Social Sharing | I encouraged them to share how they were feeling (e.g., “tell me more” |
| No Regulation | I did not try to change their emotion |

**Table 2**

*Semantic Similarity: Cosine Values*

| ER Strategy | EMA Item | PACER Item | Item cosine | Average cosine |
| --- | --- | --- | --- | --- |
| Acceptance | I expressed that it was okay to feel their emotion | I help my child understand that it’s okay to have negative feelings. | .93 | .90 |
|  |  | I help my child accept their negative feelings. | .85 |  |
|  |  | I help my child accept the way they are feelings if they are unable to change the situation causing those feelings. | .92 |  |
|  |  | I tell my child that having negative feelings is okay. | .86 |  |
|  |  | I stress to my child that it can be helpful to accept negative feelings in some situations. | .94 |  |
| Cognitive Reappraisal | Offered them ways to interpret the situation (e.g., explained reasoning) | I help my child think of a situation in a positive light. | .81 | .91 |
|  |  | I help my child see the situation from a different perspective. | .94 |  |
|  |  | I help my child try to see the positive aspects of a situation that is making them have negative feelings. | .92 |  |
|  |  | I help my child change their feelings by thinking differently about their current situation. | .92 |  |
|  |  | I encourage my child to think of the positive side to their negative feelings. | .94 |  |
| Distraction | I encouraged them to do something pleasant (e.g., watch cartoons) | I help my child find ways to distract themselves from their negative feelings. | .87 | .88 |
|  |  | I help my child distract themselves from their negative feelings by finding other things to do. | .88 |  |
|  |  | I help my child take their mind off of things that are making them have negative feelings. | .87 |  |
|  |  | I help my child take their attention off something that is making them have negative feelings. | .89 |  |
|  |  | I help my child think about something other than what is making them have negative feelings. | .89 |  |
| Expressive Suppression | I verbally encouraged them to change their emotion (i.e. “don’t cry”) | I help my child to not show their negative feelings. | .92 | .92 |
|  |  | I help my child try to hide their feelings from others. | .90 |  |
|  |  | I help my child hide their physical expressions of their negative feelings. | .87 |  |
|  |  | I help my child hide their negative feelings so that it is very hard for other people to tell how they are feeling in the moment. | .94 |  |
|  |  | I encourage my child to hide negative feelings from others. | .96 |  |

**Table 3**

*Semantic Similarity: Bivariate Correlations*

| ER Strategy | EMA Item | PACER Item | Inter-item *r* | Scale *r* |
| --- | --- | --- | --- | --- |
| Acceptance | I expressed that it was okay to feel their emotion | I help my child understand that it’s okay to have negative feelings. | .35*** | .31*** |
|  |  | I help my child accept their negative feelings. | .29*** |  |
|  |  | I help my child accept the way they are feelings if they are unable to change the situation causing those feelings. | .27*** |  |
|  |  | I tell my child that having negative feelings is okay. | .32*** |  |
|  |  | I stress to my child that it can be helpful to accept negative feelings in some situations. | .21*** |  |
| Cognitive Reappraisal | Offered them ways to interpret the situation (e.g., explained reasoning) | I help my child think of a situation in a positive light. | .07 | .04 |
|  |  | I help my child see the situation from a different perspective. | .10 |  |
|  |  | I help my child try to see the positive aspects of a situation that is making them have negative feelings. | .05 |  |
|  |  | I help my child change their feelings by thinking differently about their current situation. | .05 |  |
|  |  | I encourage my child to think of the positive side to their negative feelings. | -.06 |  |
| Distraction | I encouraged them to do something pleasant (e.g., watch cartoons) | I help my child find ways to distract themselves from their negative feelings. | .08 | .12 |
|  |  | I help my child distract themselves from their negative feelings by finding other things to do. | .13 |  |
|  |  | I help my child take their mind off of things that are making them have negative feelings. | .11 |  |
|  |  | I help my child take their attention off something that is making them have negative feelings. | .11 |  |
|  |  | I help my child think about something other than what is making them have negative feelings. | .11 |  |
| Expressive Suppression | I verbally encouraged them to change their emotion (i.e. “don’t cry”) | I help my child to not show their negative feelings. | .28*** | .30*** |
|  |  | I help my child try to hide their feelings from others. | .25** |  |
|  |  | I help my child hide their physical expressions of their negative feelings. | .29*** |  |
|  |  | I help my child hide their negative feelings so that it is very hard for other people to tell how they are feeling in the moment. | .25** |  |
|  |  | I encourage my child to hide negative feelings from others. | .30*** |  |

* *p* < .05, ** *p* < .01, *** *p* < .001

**Table 4**

Multiple Linear Regression of EMA Measures Predicting PACER Acceptance

| Predictor | *b* | *b*  95% CI  [LL, UL] | *sr^2^* | *sr^2^*  95% CI  [LL, UL] | Fit |
| --- | --- | --- | --- | --- | --- |
| (Intercept) | 32.28** | [26.08, 38.49] |  |  |  |
| EMA Acceptance | 3.48** | [1.62, 5.34] | .07 | [.00, .14] |  |
| EMA Distraction | 0.54 | [-2.15, 3.24] | .00 | [-.01, .01] |  |
| EMA Reappraisal | -0.07 | [-1.98, 1.84] | .00 | [-.00, .00] |  |
| EMA Suppression | -6.05** | [-9.43, -2.67] | .07 | [-.00, .13] |  |
| Child Age | -0.10 | [-0.67, 0.47] | .00 | [-.01, .01] |  |
| Child Gender^a^ | -0.34 | [-1.52, 0.85] | .00 | [-.01, .01] |  |
| Caregiver Gender^b^ | -1.37 | [-4.14, 1.39] | .01 | [-.01, .02] |  |
| Caregiver Edu. | -0.07 | [-0.33, 0.19] | .00 | [-.01, .01] |  |
| Child Lack of Impulse Control | 0.00 | [-0.11, 0.12] | .00 | [-.00, .00] |  |
|  |  |  |  |  | *R^2^*  = .218** |
| *F*(9, 149) = 4.6, *p* < .001 |  |  |  |  | 95% CI  [.07,.29] |

*Note.* A significant *b*-weight indicates the semi-partial correlation is also significant. *b* represents unstandardized regression weights. *sr^2^* represents the semi-partial correlation squared. *LL* and *UL* indicate the lower and upper limits of a confidence interval, respectively. EMA = ecological momentary assessment. PACER = Parental Assistance with Child Emotion Regulation (Cohodes et al., 2022). Reappraisal = Cognitive Reappraisal; Suppression = Expressive Suppression. Child Lack of Impulse Control (Integrative Child Temperament Scale, Zentner, 2020).

^a^ Child Gender coded 1 = Female, 0 = Not Female

^b^ Caregiver Gender coded 1 = Female, 0 = Not Female

^c^ Years of education completed, *z*-scored

^d^ Composite of frustration proneness and reversed attentional control subscales; higher scores indicate more child difficulty with impulse control

* indicates *p* < .05. ** indicates *p* < .01.

**Table 5**

Multiple Linear Regression of EMA Measures Predicting PACER Distraction

| Predictor | *b* | *b*  95% CI  [LL, UL] | *sr^2^* | *sr^2^*  95% CI  [LL, UL] | Fit |
| --- | --- | --- | --- | --- | --- |
| (Intercept) | 24.62** | [14.07, 35.18] |  |  |  |
| EMA Acceptance | 1.16 | [-1.87, 4.19] | .00 | [-.01, .02] |  |
| EMA Distraction | 2.99 | [-1.27, 7.25] | .01 | [-.02, .04] |  |
| EMA Reappraisal | 2.17 | [-0.93, 5.28] | .01 | [-.02, .04] |  |
| EMA Suppression | 7.32** | [2.24, 12.40] | .05 | [-.01, .11] |  |
| Child Age | -0.11 | [-1.06, 0.83] | .00 | [-.01, .01] |  |
| Child Gender^a^ | -0.33 | [-2.33, 1.67] | .00 | [-.01, .01] |  |
| Caregiver Gender^b^ | -3.55 | [-8.07, 0.97] | .01 | [-.02, .05] |  |
| Caregiver Edu.^c^ | 0.10 | [-0.34, 0.54] | .00 | [-.01, .01] |  |
| Child Lack of Impulse Control^d^ | -0.03 | [-0.23, 0.17] | .00 | [-.01, .01] |  |
|  |  |  |  |  | *R^2^*  = .120* |
| *F*(9, 144) = 2.18, *p* < .05 |  |  |  |  | 95% CI  [.00,.17] |

*Note.* A significant *b*-weight indicates the semi-partial correlation is also significant. *b* represents unstandardized regression weights. *sr^2^* represents the semi-partial correlation squared. *LL* and *UL* indicate the lower and upper limits of a confidence interval, respectively. EMA = ecological momentary assessment. PACER = Parental Assistance with Child Emotion Regulation (Cohodes et al., 2022). Reappraisal = Cognitive Reappraisal; Suppression = Expressive Suppression. Child Lack of Impulse Control (Integrative Child Temperament Scale, Zentner, 2020).

^a^ Child Gender coded 1 = Female, 0 = Not Female

^b^ Caregiver Gender coded 1 = Female, 0 = Not Female

^c^ Years of education completed, *z*-scored

^d^ Composite of frustration proneness and reversed attentional control subscales; higher scores indicate more child difficulty with impulse control

* indicates *p* < .05. ** indicates *p* < .01.

**Table 6**

*Multiple Linear Regression of EMA Measures Predicting PACER Cognitive Reappraisal*

| Predictor | *b* | *b*  95% CI  [LL, UL] | *sr^2^* | *sr^2^*  95% CI  [LL, UL] | Fit |
| --- | --- | --- | --- | --- | --- |
| (Intercept) | 29.23** | [20.44, 38.02] |  |  |  |
| EMA Acceptance | 0.03 | [-2.55, 2.61] | .00 | [-.00, .00] |  |
| EMA Distraction | -0.39 | [-4.08, 3.30] | .00 | [-.00, .01] |  |
| EMA Reappraisal | 1.65 | [-0.96, 4.26] | .01 | [-.02, .04] |  |
| EMA Suppression | 2.70 | [-1.62, 7.03] | .01 | [-.02, .04] |  |
| Child Age | 0.73 | [-0.03, 1.50] | .02 | [-.02, .07] |  |
| Child Gender^a^ | -1.14 | [-2.76, 0.48] | .01 | [-.02, .04] |  |
| Caregiver Gender^b^ | -1.96 | [-5.71, 1.79] | .01 | [-.02, .03] |  |
| Caregiver Edu.^c^ | -0.06 | [-0.43, 0.31] | .00 | [-.01, .01] |  |
| Child Lack of Impulse Control^d^ | -0.21* | [-0.37, -0.04] | .04 | [-.02, .09] |  |
|  |  |  |  |  | *R^2^*  = .105 |
| *F*(9, 145) = 1.89, *p* = .057 |  |  |  |  | 95% CI  [.00,.15] |

*Note.* A significant *b*-weight indicates the semi-partial correlation is also significant. *b* represents unstandardized regression weights. *sr^2^* represents the semi-partial correlation squared. *LL* and *UL* indicate the lower and upper limits of a confidence interval, respectively. EMA = ecological momentary assessment. PACER = Parental Assistance with Child Emotion Regulation (Cohodes et al., 2022). Reappraisal = Cognitive Reappraisal; Suppression = Expressive Suppression. Child Lack of Impulse Control (Integrative Child Temperament Scale, Zentner, 2020).

^a^ Child Gender coded 1 = Female, 0 = Not Female

^b^ Caregiver Gender coded 1 = Female, 0 = Not Female

^c^ Years of education completed, *z*-scored

^d^ Composite of frustration proneness and reversed attentional control subscales; higher scores indicate more child difficulty with impulse control

* indicates *p* < .05. ** indicates *p* < .01.

**Table 7**

Multiple Linear Regression of EMA Measures Predicting PACER Expressive Suppression

| Predictor | *b* | *b*  95% CI  [LL, UL] | *sr^2^* | *sr^2^*  95% CI  [LL, UL] | Fit |
| --- | --- | --- | --- | --- | --- |
| (Intercept) | 12.23** | [4.07, 20.40] |  |  |  |
| EMA Acceptance | -2.56* | [-4.83, -0.28] | .03 | [-.02, .07] |  |
| EMA Distraction | 0.28 | [-3.04, 3.60] | .00 | [-.00, .00] |  |
| EMA Reappraisal | 0.29 | [-2.09, 2.66] | .00 | [-.00, .01] |  |
| EMA Suppression | 8.49** | [4.06, 12.91] | .08 | [.00, .16] |  |
| Child Age | -0.16 | [-0.85, 0.52] | .00 | [-.01, .01] |  |
| Child Gender^a^ | 0.08 | [-1.40, 1.55] | .00 | [-.00, .00] |  |
| Caregiver Gender^b^ | 0.67 | [-3.46, 4.80] | .00 | [-.01, .01] |  |
| Caregiver Edu.^c^ | -0.04 | [-0.37, 0.29] | .00 | [-.00, .01] |  |
| Child Lack of Impulse Control^d^ | -0.08 | [-0.23, 0.06] | .01 | [-.02, .03] |  |
|  |  |  |  |  | *R^2^*  =  .168** |
| *F*(9, 151) = 3.38, *p* < .001 |  |  |  |  | 95% CI  [.03,.23] |

*Note.* A significant *b*-weight indicates the semi-partial correlation is also significant. *b* represents unstandardized regression weights. *sr^2^* represents the semi-partial correlation squared. *LL* and *UL* indicate the lower and upper limits of a confidence interval, respectively. EMA = ecological momentary assessment. PACER = Parental Assistance with Child Emotion Regulation (Cohodes et al., 2022). Reappraisal = Cognitive Reappraisal; Suppression = Expressive Suppression. Child Lack of Impulse Control (Integrative Child Temperament Scale, Zentner, 2020).

^a^ Child Gender coded 1 = Female, 0 = Not Female

^b^ Caregiver Gender coded 1 = Female, 0 = Not Female

^c^ Years of education completed, *z*-scored

^d^ Composite of frustration proneness and reversed attentional control subscales; higher scores indicate more child difficulty with impulse control

* indicates *p* < .05. ** indicates *p* < .01.
